# Modularity promotes morphological divergence in ray-finned fishes

**DOI:** 10.1038/s41598-018-25715-y

**Published:** 2018-05-08

**Authors:** Olivier Larouche, Miriam L. Zelditch, Richard Cloutier

**Affiliations:** 1 Department of Biological Sciences, Clemson University, Clemson, SC 29631 USA; 2 Museum of Paleontology, University of Michigan, Ann Arbor, MI 48109 USA; 3 Laboratoire de Recherche en Paléontologie et Biologie évolutive, Université du Québec à Rimouski, Rimouski, QC G5L 3A1 Canada

## Abstract

Modularity is considered a prerequisite for the evolvability of biological systems. This is because in theory, individual modules can follow quasi-independent evolutionary trajectories or evolve at different rates compared to other aspects of the organism. This may influence the potential of some modules to diverge, leading to differences in disparity. Here, we investigated this relationship between modularity, rates of morphological evolution and disparity using a phylogenetically diverse sample of ray-finned fishes. We compared the support for multiple hypotheses of evolutionary modularity and asked if the partitions delimited by the best-fitting models were also characterized by the highest evolutionary rate differentials. We found that an evolutionary module incorporating the dorsal, anal and paired fins was well supported by the data, and that this module evolves more rapidly and consequently generates more disparity than other modules. This suggests that modularity may indeed promote morphological disparity through differences in evolutionary rates across modules.

## Introduction

Biological systems can be subdivided into highly integrated component parts called modules^1–3^. These modules are discrete and internally coherent units that develop or evolve quasi-independently from other parts of the organism^4–6^. The modular organization of biological systems is hypothesized to facilitate evolvability^1,7–9^: given their quasi-independence, individual modules can be modified without interfering with other such modules^10–12^. This is not to say that hypothetical non-modular organisms cannot evolve, simply that more modular organisms are expected to evolve more rapidly and/or in more directions^13–16^. Modularity can also enhance differences in evolutionary rates among traits^13,17,18^ and those rate differentials, in turn, can determine the extent to which lineages may diverge morphologically, leading to differences in disparity among evolving clades^19–24^. Another hypothesis, however, is that modularity can constrain morphological evolution, thereby decreasing evolutionary rates^10,25,26^.

Among actinopterygians or ray-finned fishes (~30 000 living species), there is a well-known evolutionary trend for shifts to occur in the size and relative position of both the median and paired fins^27–29^. These changes in size and position contribute to the impressively high disparity in fin configuration patterns that is observed in actinopterygians^30^. Here, we demonstrate that this evolutionary lability (i.e., a greater potential to generate phenotypic variation on an evolutionary timescale) in fin configurations could be related to a modular organization of fish appendages, resulting in relatively higher rates of morphological evolution and therefore higher disparity for median and paired fins modules.

## Results

### Phylomorphospace analysis

We constructed a phylomorphospace of shape data to investigate the patterns of morphological disparity among seven actinopterygian subgroups. The phylomorphospace analysis reconstructs ancestral shapes, including them as well as the shapes of living species in a principal component analysis (PCA) and projecting the phylogeny onto that space^23,31^.

**Figure 1:**
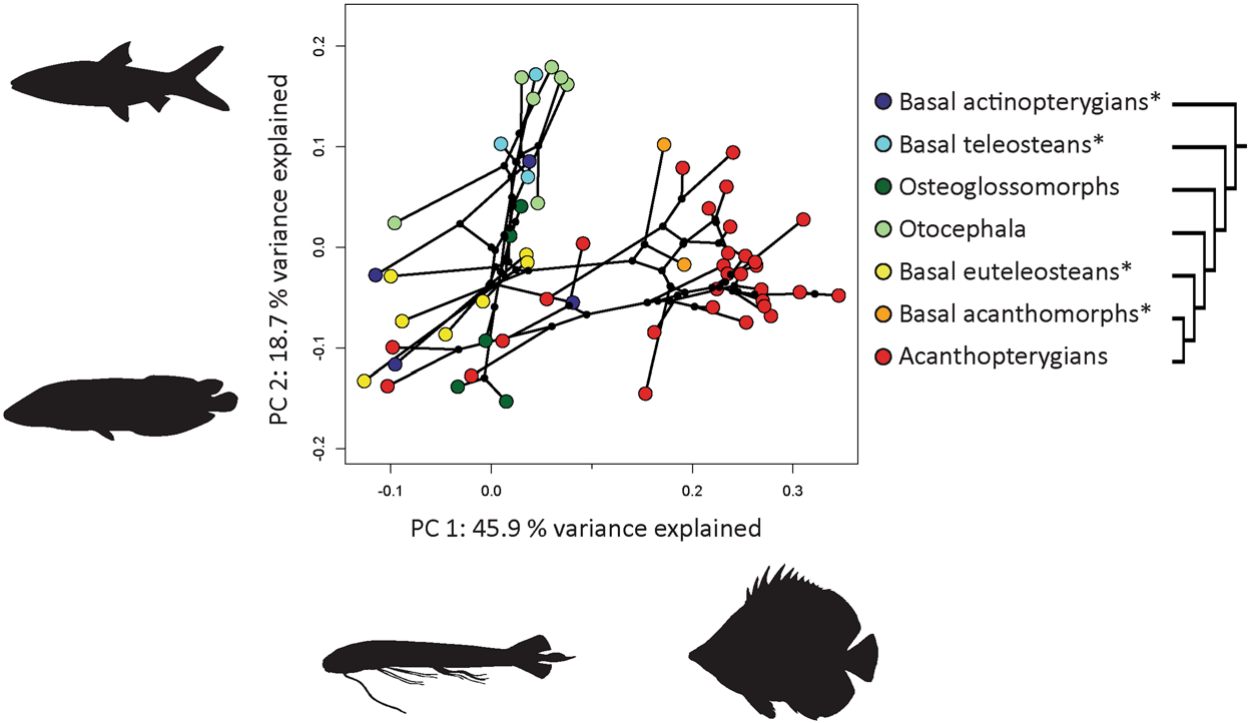
Phylomorphospace analysis of the body shape of 58 actinopterygian species. Fish silhouettes represent the most extreme forms along the first and second axes of the PCA. The simplified phylogeny summarizes the molecular phylogenetic hypothesis from Near *et al*.^50^. Groups marked with an asterisk are the basal members of their respective clades and are thus paraphyletic.

The first axis of the phylomorphospace (Fig. 1, Supplementary Fig. S1) strikingly contrasts acanthomorphs, a large clade of derived actinopterygians characterized by the presence of spines along the leading edges of the dorsal and anal fins, to all other more basal actinopterygians. In terms of body shapes and fin configurations, the first axis contrasts fishes with a more fusiform shape where the dorsal, anal and pelvic fins are placed more posteriorly, to deeper forms where the dorsal, anal and pelvic fins migrate or extend anteriorly. The first axis also highlights an evolutionary trend among derived actinopterygians for the dorsal and anal fins to have more elongated bases. A few acanthopterygians stand out as exceptions to these patterns and share the morphospace of other more basal actinopterygians (i.e. some members of the Cyprinodontiformes, Beloniformes and Cetomimiformes). The second axis opposes one group comprising basal teleosteans and most of the ostarioclupeomorphs to a second group comprising basal euteleosteans, most of the acanthomorphs as well as some osteoglossomorphs. On this axis, the contrast in body shapes and fin configurations opposes morphologies where the caudal peduncle is long and narrow, to morphologies where the dorsal and anal fins are more elongated and where the caudal peduncle is shorter. Additionally, in the former case the pelvic fins are inserted approximately at the middle of the antero-posterior body axis, whereas in the latter case they migrate anteriorly.

The phylomorphospace emphasizes the importance of changes in fin configurations as a major evolutionary trend driving patterns of morphological disparity among actinopterygians. Next, we investigate how modularity may influence these disparity patterns by allowing differing rates of morphological evolution among modules.

### Evolutionary modularity

We identified the five best-supported hypotheses for evolutionary modularity based on the best-fitting models from the graphical modeling and minimum deviance approaches (Table 1). Overall, two hypotheses of modularity are well supported by both criteria for model selection: AIC and γ*. Hypothesis 3 subdivides the fish’s body into two modules: (1) the tail (caudal peduncle and fin) and (2) the head plus trunk (dorsal, anal and paired fins). Hypothesis 5 further subdivides the body into three modules: (1) head, (2) trunk, and (3) tail. Despite a larger difference in AIC, hypothesis 4 is also well-supported by graphical modeling. All three of the best-supported hypotheses of evolutionary modularity based on graphical modeling share the expectation that dorsal, anal and paired fins are integrated with one-another; they differ as to whether these fins are also integrated with the head or tail regions.

**Table 1:** Results of the statistical analyses of modularity using graphical modeling, minimum deviance and covariance ratio methods.

Hypothesis number	Modules	Graphical modeling		Minimum deviance		Covariance ratio	
		Deviance	∆AIC	γ*	***p***	CR	***p***
1	head/all fins/tail peduncle	70.03	34.93	−0.416	0.936	0.8674	0.005
2	head/paired fins/dorsal and anal fins/tail peduncle/caudal fin	115.43	70.33	−0.394	0.334	0.9914	0.015
3	head and trunk/tail	31.10	0	−0.376	1	0.9705	0.125
4	head/trunk and tail	26.17	7.07	−0.230	1	0.8268	0.01
5	head/trunk/tail	37.99	0.89	−0.375	0.92	0.8697	0.004

Two additional hypotheses were strongly supported by the minimum deviance method but less so by graphical modeling. Hypothesis 1, like the best-fitting hypotheses from graphical modeling, predicts that dorsal, anal and paired fins are integrated, but this fin module also includes the caudal fin. Hypothesis 2 subdivides the fins into three modules: (1) paired, (2) dorsal and anal and (3) caudal. Perhaps this hints towards the hierarchical nature of modularity, whereby smaller fin modules can be nested within larger ones.

Four of the five hypotheses of evolutionary modularity yield statistically significant covariance ratios, even though CR values are relatively high. For hypotheses 1, 2 and 5, the matrices of pairwise CR values provide additional insight into patterns of integration and modularity (Supplementary Tables S2, S3 and S4). Notably, the CR values between parts of the tail (caudal fin and peduncle) and other partitions are generally low. Moreover for hypothesis 2, the high CR value between the dorsal/anal and paired fins indicates that these fins are strongly integrated, supporting an evolutionary module comprising the fins inserted along the trunk region.

### Evolutionary rates and disparity among morphological partitions

**Figure 2:**
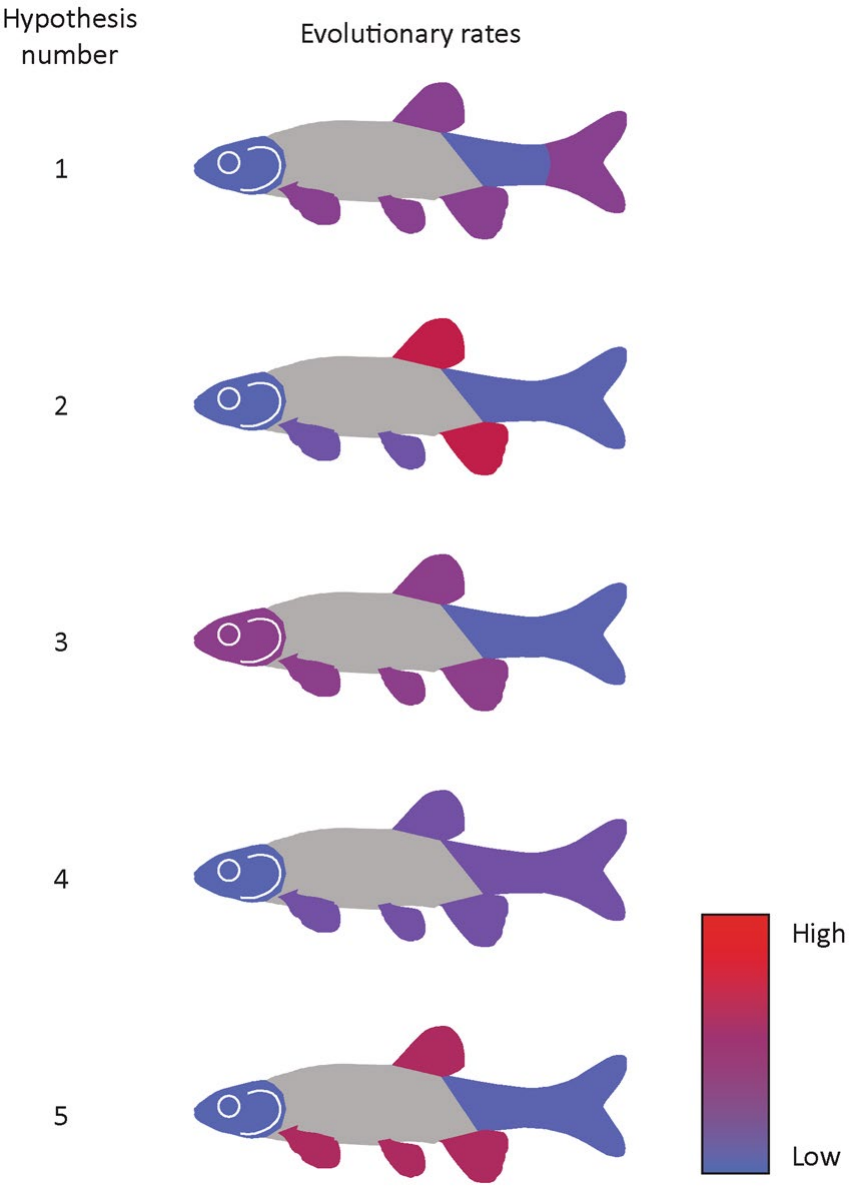
Evolutionary rate estimates for the partitions of the five best-fitting modularity hypotheses. Shades of blue represent lower evolutionary rates, whereas shades of red represent higher evolutionary rates.

We used the evolutionary rate ratios to determine if evolutionary modules identified by graphical modeling and the minimum deviance approaches evolved at different rates. Two of the five best-fitting hypotheses of body shape evolutionary modularity are also well supported as evolutionary rate modules, owing to their significant rate ratios. These are the same two hypotheses that are well-supported by both graphical modeling and the minimum deviance (Table 2, Fig. 2). Additionally, the trunk region, when treated as an evolutionary module distinct from the head and tail, has a rate of morphological evolution more than five times higher than that of the tail region, and more than three times higher than that of the head region and is accordingly more disparate than these two regions (Supplementary Table S5).

**Table 2:** Results of the evolutionary rate ratios.

Hypothesis number	Modules	Rate ratio	
		ratio	*p*
1	head/all fins/tail peduncle	3.324	0.192
2	head/paired fins/dorsal and anal fins/tail peduncle/caudal fin	6.173	0.962
3	head and trunk/tail	3.547	0.001
4	head/trunk and tail	1.360	1
5	head/trunk/tail	5.174	0.001

## Discussion

Modularity is expected to allow individual parts of an organism to evolve quasi-independently^4–6^, which could then lead to differences in evolutionary rates across these parts. One consequence is that some modules can diverge more rapidly than others and therefore generate more disparity. This relationship between differential evolutionary rates and disparities across modules is well supported by our findings that the two hypotheses of evolutionary modularity that are best-supported across methods also yield significantly different evolutionary rates for modules. Furthermore, the putative evolutionary modules that incorporate median and/or paired fins have higher evolutionary rates and are consequently more disparate. The importance in evolutionary changes in median and paired fins morphologies in generating the disparity among actinopterygians is further emphasized by the first principal axis of the phylomorphospace analysis, contrasting acanthomorphs to other more basal actinopterygians.

Despite our results, the relationship between modularity, evolutionary rate differentials and disparity is likely to be complex. One hypothesis is that increased modularity may favor higher evolutionary rates as well as increased disparity. This is consistent with the results of an investigation focusing on Mantis shrimp raptorial appendages documenting higher rates of phenotypic evolution for groups with higher degrees of modularity^32^. This is also consistent with a study focusing on functional integration of the feeding apparatus in eels (Anguilliformes) that showed that reduced integration (or increased modularity) of the components in biting eels yields higher levels of disparity when compared to the suction-feeding eels^33^. In contrast, no relationship between disparity and overall level of integration was found in crinoids^34^. Another hypothesis is that disparity could be related to the level of integration of individual modules, meaning that highly integrated modules might be more constrained. For instance in mammalian^35^ and bird^36^ crania, modules with lower integration were found to be generally more disparate than those with stronger integration. On the other hand, a relationship between all three of these components (modularity, evolutionary rates and disparity) should not always be expected. Differences in disparity do not consistently coincide with differences in evolutionary rates across modules^33,37,38^. One possible explanation is that highly integrated modules can influence disparity by favouring the evolution of integrated traits along a number of preferred directions, but without affecting evolutionary rates^37^.

Here we propose that at least in ray-finned fishes, higher rates of morphological evolution for the fins inserted along the trunk region may have contributed to the extensive disparity in fin configurations characteristic of the more advanced actinopterygians during their evolutionary diversification. Indeed, the analyses of evolutionary modularity emphasize the importance of the dorsal, anal and paired fins integration patterns. Furthermore, the high disparity in the trunk region, taken together with the higher evolutionary rates, are consistent with the idea that modularity can indeed enhance disparity by allowing for differing rates of morphological evolution among modules^17,18^. Our finding that the rate of trunk evolution is high relative to that of the head in fishes is consistent with a study of lantern fishes (Myctophiformes)^17^. Despite some differences in landmark sampling schemes between that study and ours, as well as the difference in phylogenetic scope, these higher rates of morphological evolution for the trunk region could reflect the known evolutionary trends among actinopterygians for shifts to occur in the size and relative position of both median and paired fins^27–29^. Thus, our results support the idea that evolutionary modularity can promote morphological divergence in diverse directions by allowing different rates of phenotypic evolution among modules. Modularity may not always suffice to yield differential evolutionary rates across modules; the face and braincase may be modules in both gymnotiform fishes and carnivoran mammals, for example, but only carnivoran mammals have been found to exhibit significant differences in evolutionary rates between those modules^18^. Because modularity permits but does not ensure that rates of morphological evolution differ across modules, the strong connection that we find here between modularity, morphological disparity and differential evolutionary rates may be just one of the possible outcomes of a modular organization.

## Methods

### Shape analysis

**Figure 3:**
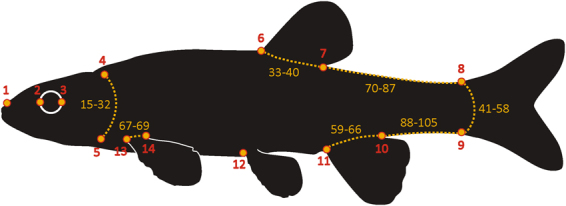
Positioning of landmarks (numbered in red) and semi-landmarks (numbered in yellow).

Hypotheses of evolutionary modularity were examined using geometric morphometric data. The sample comprised 58 actinopterygian species covering a wide phylogenetic spectrum from basal actinopterygians (e.g., sturgeons and bowfins) to derived acanthopterygians (Supplementary Table S6). We assembled a dataset of photographs of specimens from online repositories of catalogued collections, with some additional photographs taken while visiting museum collections. Because all specimens are required to have the same number of homologous landmarks, all specimens shared the same general fin configuration (i.e. single dorsal and anal fins, caudal fin, pectoral and pelvic fins). To analyze fin-positioning, nine landmarks were positioned at the fin insertion points and five additional landmarks were positioned at the anterior limit of the rostrum, anterior and posterior limits of the eye and dorsal and ventral limits of the gill slit (Fig. 3). Semi-landmarks were used to provide additional information along curves of the body where landmarks could not be consistently positioned in homologous locations. A total of 55 semi-landmarks were placed along the outline of the operculum, the base of each fin and the outline of the body between the posterior end of the dorsal fin and the dorsal insertion of the caudal fin, and between the posterior end of the anal fin and the ventral insertion of the caudal fin. Digitized coordinates were superimposed using a General Procrustes Analysis^39^ and semi-landmarks were superimposed using the minimum bending energy criterion^40,41^.

### Testing hypotheses of modularity

A total of 24 *a priori* hypotheses of modularity were analyzed (Supplementary Table S7). Three approaches were used to test these hypotheses: (1) the covariance ratio (CR) which is a measure of the relative strength of associations among subsets of landmarks compared to associations across these subsets^42^, (2) the analysis of correlations between shapes of subsets of landmarks using graphical modeling^5,43–46^, (3) and the standardized gamma statistic (γ*) which is a measure of the goodness-of-fit of the covariance matrix derived from a model to the data^47,48^.

### Comparing evolutionary rates and disparity among modules

To determine whether rates differed among partitions, we used the evolutionary rate ratio^17^, which is a ratio between multivariate rates of morphological evolution^49^. Rates are estimated for each hypothesized module and compared to each other by generating a null distribution of rate ratios, obtained by simulating datasets along the phylogeny using a single rate Brownian motion model^17,49^. Disparity was measured as the Procrustes variance, calculated for each partition. Following the method for measuring evolutionary rates, disparity was scaled for the number of landmarks by subdividing the result by that number.

### Phylogenetic context

The phylogenetic context for all of our analyses (excepting the minimum deviance method) was provided by a pruned version of a large-scale time-calibrated actinopterygian phylogeny based on the partitioned maximum-likelihood analysis of nine nuclear gene sequences^50^. However, the minimum deviance method does not currently allow to take into account phylogenetic signal. We compared the evolutionary rate matrix to the non-phylogenetic covariance matrix and obtained a very high correlation between the two matrices (r = 0.908, *p* < 0.0001), indicating that both matrices are structurally very similar. Thus, we considered that the minimum deviance method could be used even though a procedure currently does not exist to account for phylogeny.

### Software used to perform the analyses

Landmarks and semi-landmarks were digitized with TPSDig2^51^ and superimposed using the package geomorph^52^ for R^53^. The phylomorphospace analysis, covariance ratio and rate ratio were also computed using the package geomorph^52^. The observed correlation matrices for the distance matrix-based method were generated using a script developed by Adam Rountrey [available as supplementary material for Zelditch *et al*.^54^, at http://booksite.elsevier.com/9780123869036/functions.php], and modified by M.L.Z. to account for phylogeny. Graphical modeling was performed in R using the package ggm^55^. The minimum deviance method was implemented using the program Mint version 1.61^56^.

### Data availability

The datasets analyzed during the current study are available from the corresponding author on reasonable request.

## Electronic supplementary material

Supplementary Information

## References

[1] Wagner GP (1996). Homologues, natural kinds and the evolution of modularity. Am. Zool..

[2] Winther RG (2001). Varieties of modules: Kinds, levels, origins, and behaviors. J. Exp. Zool..

[3] Klingenberg CP (2008). Morphological integration and developmental modularity. Annu. Rev. Ecol. Evol. Syst..

[4] Simon HA (1962). The architecture of complexity. Proc. Amer. Phil. Soc..

[5] Magwene PM (2001). New tools for studying integration and modularity. Evolution.

[6] Müller GB (2007). Evo-devo: Extending the evolutionary synthesis. Nat. Rev. Genet..

[7] Bonner, J. T. *The evolution of complexity by means of natural selection*. (Princeton University Press, 1988).

[8] Raff, R. A. *The Shape of Life: Genes, Development, and the Evolution of Animal Form*. (The University of Chicago Press, 1996).

[9] Wagner GP, Altenberg L (1996). Perspective: Complex adaptations and the evolution of evolvability. Evolution.

[10] Hansen TF (2003). Is modularity necessary for evolvability? Remarks on the relationship between pleiotropy and evolvability. Biosystems.

[11] Hansen TF (2006). The evolution of genetic architecture. Annu. Rev. Ecol. Evol. Syst..

[12] Hansen TF, Armbruster WS, Carlson ML, Pelabon C (2003). Evolvability and genetic constraint in *Dalechampia* blossoms: Genetic correlations and conditional evolvability. J. Exp. Zool. Part B.

[13] West-Eberhard, M. J. *Developmental Plasticity and Evolution*. (Oxford University Press, 2003).

[14] Simon, H. A. The organization of complex systems in *Hierarchy Theory: The Challenge of Complex* Systems (ed. Pattee, H. H.) 3–27 (George Braziller, 1973).

[15] Larsen, E. W. Evolution of development: The shuffling of ancient modules by ubiquitous bureaucracies in *Physical Theory in Biology: Foundations and Explorations* (eds Lumsden, C. J., Trainor, L. E. H. & Brandts, W. A.) 431–441 (World Scientific Publishing, 1997).

[16] Jablonski D (2017). Approaches to macroevolution: 1. General concepts and origin of variation. Evol. Biol..

[17] Denton JSS, Adams DC (2015). A new phylogenetic test for comparing multiple high-dimensional evolutionary rates suggests interplay of evolutionary rates and modularity in lanternfishes (Myctophiformes; Myctophidae). Evolution.

[18] Evans KM, Waltz BT, Tagliacollo VA, Sidlauskas BL, Albert JS (2017). Fluctuations in evolutionary integration allow for big brains and disparate faces. Sci. Rep..

[19] Foote M (1997). The evolution of morphological diversity. Annu. Rev. Ecol. Syst..

[20] Harmon LJ, Schulte JA, Larson A, Losos JB (2003). Tempo and mode of evolutionary radiation in iguanian lizards. Science.

[21] Ackerly DD, Nyffeler R (2004). Evolutionary diversification of continuous traits: Phylogenetic tests and application to seed size in the California flora. Evol. Ecol..

[22] O’Meara BC, Ané C, Sanderson MJ,  Wainwright PC (2006). Testing for different rates of continuous trait evolution using likelihood. Evolution.

[23] Sidlauskas B (2008). Continuous and arrested morphological diversification in sister clades of characiform fishes: A phylomorphospace approach. Evolution.

[24] Sidlauskas B (2007). Testing for unequal rates of morphological diversification in the absence of a detailed phylogeny: A case study from characiform fishes. Evolution.

[25] Wagner GP (1984). Coevolution of functionally constrained characters: Prerequisites for adaptive versatility. BioSystems.

[26] Wagner GP (1988). The influence of variation and of developmental constraints on the rate of multivariate phenotypic evolution. J. Evol. Biol..

[27] Lauder GV, Drucker EG (2004). Morphology and experimental hydrodynamics of fish fin control surfaces. IEEE J. Ocean. Eng..

[28] Lauder GV, Liem KF (1983). The evolution and interrelationships of the actinopterygian fishes. Bull. Mus. Comp. Zool..

[29] Webb PW (1982). Locomotor patterns in the evolution of actinopterygian fishes. Am. Zool..

[30] Larouche O, Zelditch ML, Cloutier R (2017). Fin modules: An evolutionary perspective on appendage disparity in basal vertebrates. BMC Biol..

[31] Rohlf, F. J. Geometric morphometrics and phylogeny in *Morphology*, *Shape and Phylogeny* (eds MacLeod N. & Forey, P. L.) 175–193 (CRC Press, 2002).

[32] Claverie T, Patek SN (2013). Modularity and rates of evolutionary change in a power-amplified prey capture system. Evolution.

[33] Collar DC, Wainwright PC, Alfaro ME, Revell LJ, Mehta RS (2014). Biting disrupts integration to spur skull evolution in eels. Nat. Commun..

[34] Gerber S (2013). On the relationship between the macroevolutionary trajectories of morphological integration and morphological disparity. PLoS One.

[35] Goswami A, Polly PD (2010). The influence of modularity on cranial morphological disparity in Carnivora and Primates (Mammalia). PLoS One.

[36] Felice RN, Goswami A (2018). Developmental origins of mosaic evolution in the avian cranium. Proc. Natl. Acad. Sci. USA.

[37] Goswami A, Smaers JB, Soligo C, Polly PD (2014). The macroevolutionary consequences of phenotypic integration: From development to deep time. Philos. Trans. R. Soc. B-Biol. Sci..

[38] Linde-Medina M, Boughner JC, Santana SE, Diogo R (2016). Are more diverse parts of the mammalian skull more labile?. Ecol. Evol..

[39] Rohlf FJ, Slice D (1990). Extensions of the Procrustes method for the optimal superimposition of landmarks. Syst. Zool..

[40] Green, W. D. K. The thin-plate spline and images with curving features in *Proceedings in Image Fusion and Shape Variability Techniques* (eds Mardia, K. V., Gill, C. A. & Dryden, I. L.) 79–87 (Leeds University Press, 1996).

[41] Bookstein FL (1997). Landmark methods for forms without landmarks: Morphometrics of group differences in outline shape. Med. Image Anal..

[42] Adams DC (2016). Evaluating modularity in morphometric data: Challenges with the RV coefficient and a new test measure. Methods Ecol. Evol..

[43] Whittaker, J. *Graphical models in applied mathematical multivariate statistics*. (John Wiley and Sons, 1990).

[44] Lauritzen, S. L. *Graphical Models*. (Clarendon Press, 1996).

[45] Edwards, D. *Introduction to graphical modelling. Second edition*. (Springer-Verlag, 2000).

[46] Magwene PM (2009). Statistical methods for studying modularity: A reply to Mitteroecker and Bookstein. Syst. Biol..

[47] Richtsmeier, J. T., Lele, S. R. & Cole, T. M. III. Landmark morphometrics and the analysis of variation in *Variation: A Central Concept in Biology* (eds Hallgrímsson, B. & Hall, B. K.) 49–69 (Elsevier Academic Press, 2005).

[48] Márquez EJ (2008). A statistical framework for testing modularity in multidimensional data. Evolution.

[49] Adams DC (2014). Quantifying and comparing phylogenetic evolutionary rates for shape and other high-dimensional phenotypic data. Syst. Biol..

[50] Near TJ (2012). Resolution of ray-finned fish phylogeny and timing of diversification. Proc. Natl. Acad. Sci. USA.

[51] Rohlf, F. J. tpsDig v. 2.17 (Stony Brook University, NY, 2013).

[52] Adams DC, Otarola-Castillo E (2013). geomorph: An R package for the collection and analysis of geometric morphometric shape data. Methods Ecol. Evol..

[53] R Core Team. R: A language and environment for statistical computing v. 3.2.4 (R Foundation for Statistical Computing, Vienna, Austria, 2016).

[54] Zelditch, M. L., Swiderski, D. L. & Sheets, H. D. *Geometric Morphometrics for Biologists: A Primer. Second edition*. (Elsevier Academic Press, 2012).

[55] Marchetti, G. M., Drton, M. & Sadeghi, K. ggm: A package for graphical Markov models v. 2.0 (2014).

[56] Márquez, E. J. Mint: Modularity and integration analysis tool for morphometric data v. 1.61 (2014).

