# Supplementary material for: Modularity promotes morphological divergence in ray-finned fishes
Source: Sci Rep. 2018 May 8;8:7278. doi: 10.1038/s41598-018-25715-y (PMC5940925; doi:10.1038/s41598-018-25715-y)
Supplement: Supplementary file 1 — Supplementary Information [file 41598_2018_25715_MOESM1_ESM.pdf]

Modularity promotes morphological divergence in ray-finned fishes

OLIVIER LAROUCHE, MIRIAM L. ZELDITCH AND RICHARD CLOUTIER

Supplementary material

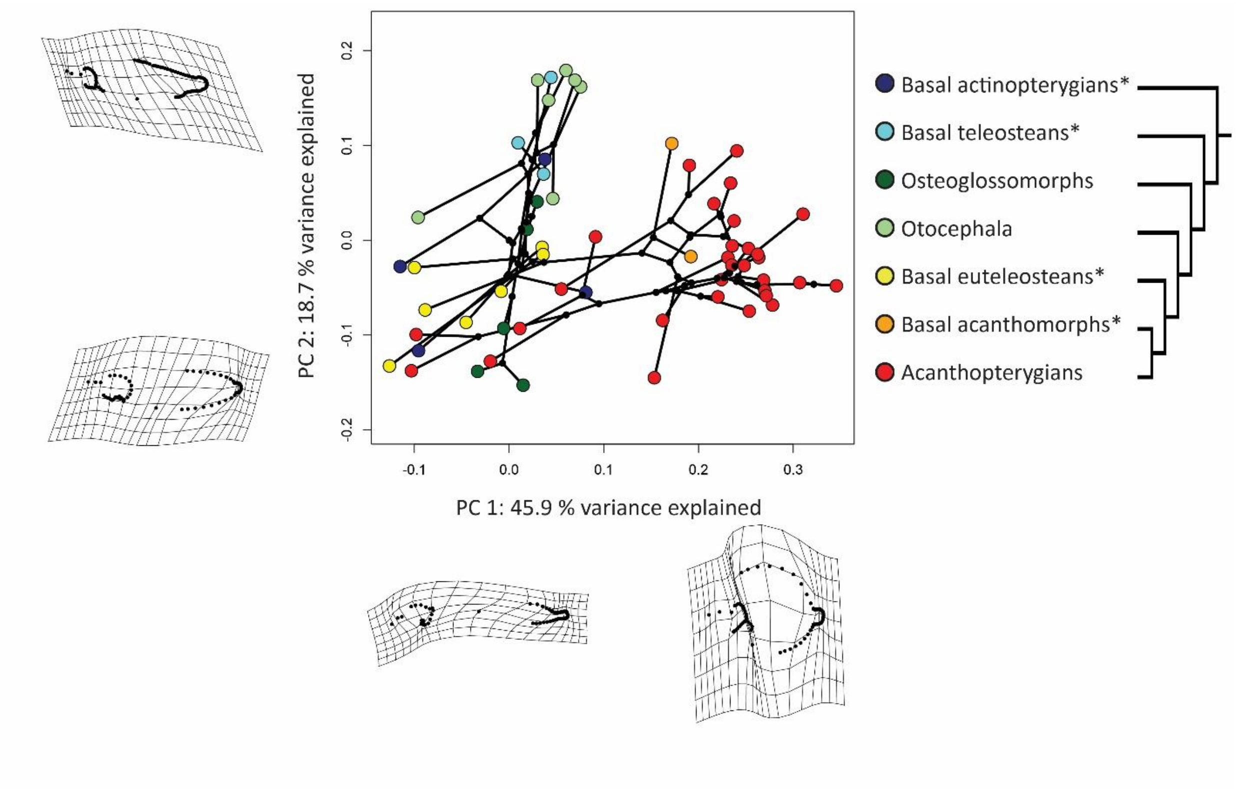

Supplementary Figure S1: Phylomorphospace analysis of the body shape of 58 actinopterygian species. Deformation grids represent the differences in shape between the extreme forms for each axis and the average shape based on the entire sample. The simplified phylogeny summarizes the molecular phylogenetic hypothesis from Near *et al.* (2012). Groups marked with an asterisk are the basal members of their respective clades and are thus paraphyletic.

Supplementary Table S2: Pairwise CR matrix for hypothesis 1.

	head	all fins
all fins	0.9164	
tail peduncle	0.8428	0.9882

Supplementary Table S3: Pairwise CR matrix for hypothesis 2.

	head	paired fins	dorsal and anal fins	tail peduncle
paired fins	0.9735			
dorsal and anal fins	0.9795	1.2709		
tail peduncle	0.8413	1.0774	1.0606	
caudal fin	0.8481	0.8413	0.9806	0.8693

Supplementary Table S4: Pairwise CR matrix for hypothesis 5.

	head	trunk
trunk	0.9079	
tail	0.8658	0.9767

Supplementary Table S5: Evolutionary rates and morphological disparity (Procrustes variance) for each of the partitions.

evolutionary modules	rate	disparity
head	7.53E-07	2.56E-04
all fins	1.48E-06	5.99E-04
tail peduncle	4.45E-07	1.73E-04
head	7.53E-07	2.56E-04
dorsal and anal fins	2.68E-06	1.09E-03
caudal fin	4.34E-07	1.54E-04
paired fins	9.55E-07	4.53E-04
tail peduncle	4.45E-07	1.73E-04
head and trunk	1.56E-06	6.19E-04
tail	4.41E-07	1.66E-04
head	7.53E-07	2.56E-04
trunk and tail	1.02E-06	4.12E-04
head	7.53E-07	2.56E-04
trunk	2.28E-06	9.41E-04
tail	4.41E-07	1.66E-04

Supplementary Table S6: List of actinopterygian species sampled.

1	Acipenser oxyrinchus	Acipenseridae	Basal Actinopterygii
2	Polyodon spathula	Polyodontidae	Basal Actinopterygii
3	Albula glossodonta	Albulidae	Basal Teleostei
4	Amia calva	Amiidae	Basal Actinopterygii
5	Oryzias hubbsi	Adrianichthyidae	Acanthopterygii
6	Strongylura strongylura	Belonidae	Acanthopterygii
7	Hyporhamphus acutus	Hemiramphidae	Acanthopterygii
8	Beryx decadactylus	Berycidae	Acanthopterygii
9	Holocentrus adscensionis	Holocentridae	Acanthopterygii
10	Ostichthys kaianus	Holocentridae	Acanthopterygii
11	Aulotrachichthys heptalepis	Trachichthyidae	Acanthopterygii
12	Gephyroberyx darwini	Trachichthyidae	Acanthopterygii
13	Hoplostethus cadenati	Trachichthyidae	Acanthopterygii
14	Barbourisia rufa	Barbourisiidae	Acanthopterygii
15	Alosa fallax	Clupeidae	Otocephala
16	Catostomus wigginsi	Catostomidae	Otocephala
17	Danio albolineatus	Cyprinidae	Otocephala
18	Pachypanchax omalonotus	Aplocheilidae	Acanthopterygii
19	Gambusia nobilis	Poeciliidae	Acanthopterygii
20	Elops hawaiensis	Elopidae	Basal Teleostei
21	Megalops atlanticus	Megalopidae	Basal Teleostei
22	Esox americanus	Esocidae	Basal Euteleostei
23	Umbra limi	Umbridae	Basal Euteleostei
24	Chanos chanos	Chanidae	Otocephala
25	Gonorynchus greyi	Gonorynchidae	Otocephala
26	Kneria wittei	Kneriidae	Otocephala
27	Atractosteus spatula	Lepisosteidae	Basal Actinopterygii
28	Ogilbia mccoskeri	Bythitidae	Acanthopterygii
29	Alepocephalus bairdii	Alepocephalidae	Basal Euteleostei

30	Galaxias maculatus	Galaxiidae	Basal Euteleostei
31	Microstoma microstoma	Microstomatidae	Basal Euteleostei
32	Holtbyrnia macrops	Platytroctidae	Basal Euteleostei
33	Arapaima gigas	Arapaimidae	Osteoglossomorpha
34	Heterotis niloticus	Arapaimidae	Osteoglossomorpha
35	Hiodon alosoides	Hiodontidae	Osteoglossomorpha
36	Hiodon tergisus	Hiodontidae	Osteoglossomorpha
37	Marcusenius ntemensis	Mormyridae	Osteoglossomorpha
38	Paracanthurus hepatus	Acanthuridae	Acanthopterygii
39	Meiacanthus grammistes	Bleniidae	Acanthopterygii
40	Enneacanthus gloriosus	Centrarchidae	Acanthopterygii
41	Chaetodon modestus	Chaetodontidae	Acanthopterygii
42	Prognathodes carlhubbsi	Chaetodontidae	Acanthopterygii
43	Cyprichromis leptosoma	Cichlidae	Acanthopterygii
44	Coris nigrotaenia	Labridae	Acanthopterygii
45	Pseudodax moluccanus	Labridae	Acanthopterygii
46	Brockius striatus	Labrisomidae	Acanthopterygii
47	Leiognathus equulus	Leiognathidae	Acanthopterygii
48	Chromis ternatensis	Pomacentridae	Acanthopterygii
49	Stegastes nigricans	Pomacentridae	Acanthopterygii
50	Epinephelus howlandi	Serranidae	Acanthopterygii
51	Siganus doliatus	Siganidae	Acanthopterygii
52	Toxotes jaculatrix	Toxotidae	Acanthopterygii
53	Aphredoderus sayanus	Aphredoderidae	Basal Acanthomorpha
54	Polymixia japonica	Polymixiidae	Basal Acanthomorpha
55	Sebastes mentella	Sebastidae	Acanthopterygii
56	Nematogenys inermis	Nematogenyidae	Otocephala
57	Poromitra crassiceps	Melamphaidae	Acanthopterygii
58	Bathophilus flemingi	Stomiidae	Basal Euteleostei

Supplementary Table S7: Description of the 24 *a priori* hypotheses of modularity tested. The numbers in parentheses refer to the landmarks and semi-landmarks included in each partition.

Hypothesis	Description of modular partitions
**1**	head + paired fins (1:5, 12:32, 67:69)<br>median fins + tail (6:11, 33:66, 70:105)
**2**	head + paired fins + tail (1:5, 12:32, 67:105)<br>median fins (6:11, 33:66)
**3**	head + caudal fin + paired fins + tail (1:5, 8:9, 12:14, 15:32, 41:58, 67:105)<br>dorsal + anal fins (6:7, 10:11, 33:40, 59:66)
**4**	head + pectoral fin (1:5, 13:14, 15:32, 67:69)<br>median fins + pelvic fin + tail (6:12, 33:66, 70:105)
**5**	head (1:5, 15:32)<br>fins (6:14, 33:69)<br>tail (70:105)
**6**	head + tail (1:5, 15:32, 70:105)<br>median fins (6:11, 33:66)<br>paired fins (12:14, 67:69)
**7**	head + tail + caudal fin (1:5, 8:9, 15:32, 41:58, 70:105)<br>dorsal + anal fins (6:7, 10:11, 33:40, 59:66)<br>paired fins (12:14, 67:69)
**8**	head + tail + caudal fin (1:5, 8:9, 15:32, 41:58, 70:105)<br>dorsal fin (6:7, 33:40)<br>ventral fins (10:14, 59:69)
**9**	head (1:5, 15:32)<br>median fins + tail (6:11, 33:66, 70:105)<br>paired fins (12:14, 67:69)
**10**	head (1:5, 15:32)<br>median fins (6:11, 33:66)<br>paired fins (12:14, 67:69)<br>tail (70:105)
**11**	head + tail + caudal fin (1:5, 8:9, 15:32, 41:58, 70:105)<br>dorsal fin (6:7, 33:40)<br>anal fin (10:11, 59:66)<br>paired fins (12:14, 67:69)

Hypothesis	Description of modular partitions
**12**	head (1:5, 15:32)<br>dorsal + anal fins (6:7, 10:11, 33:40, 59:66)<br>tail + caudal fin (8:9, 41:58, 70:105)<br>paired fins (12:14, 67:69)
**13**	head (1:5, 15:32)<br>dorsal fin (6:7, 33:40)<br>tail + caudal fin (8:9, 41:58, 70:105)<br>ventral fins (10:14, 59:69)
**14**	head + tail (1:5, 15:32, 70:105)<br>dorsal fin (6:7, 33:40)<br>caudal fin (8:9, 41:58)<br>ventral fins (10:14, 59:69)
**15**	head + tail (1:5, 15:32, 70:105)<br>dorsal fin (6:7, 33:40)<br>caudal fin (8:9, 41:58)<br>anal fin (10:11, 59:66)<br>paired fins (12:14, 67:69)
**16**	head (1:5, 15:32)<br>dorsal fin (6:7, 33:40)<br>tail + caudal fin (8:9, 41:58, 70:105)<br>anal fin (10:11, 59:66)<br>paired fins (12:14, 67:69)
**17**	head (1:5, 15:32)<br>dorsal + anal fins (6:7, 10:11, 33:40, 59:66)<br>caudal fin (8:9, 41:58)<br>paired fins (12:14, 67:69)<br>tail (70:105)
**18**	head (1:5, 15:32)<br>dorsal fin (6:7, 33:40)<br>caudal fin (8:9, 41:58)<br>anal fins (10:11, 59:66)<br>paired fins (12:14, 67:69)<br>tail (70:105)

Hypothesis	Description of modular partitions
**19**	head (1:5, 15:32)<br>dorsal (6:7, 33:40)<br>caudal fin (8:9, 41:58)<br>anal fins (10:11, 59:66)<br>paired fins (12:14, 67:69)<br>upper tail (70:87)<br>lower tail (88:105)
**20**	lower caudal peduncle (88:105)<br>all other landmarks (1:87)
**21**	head + median fins + paired fins (1:69)<br>caudal peduncle (70:105)
**22**	head + trunk (1:7, 10:40, 59:69)<br>caudal peduncle and fin (8,9, 41:58, 70:105)
**23**	head (1:5, 15:32)<br>all other landmarks (6:14, 33:105)
**24**	head (1:5, 15:32)<br>median and paired fins excluding caudal (6,7,10:14, 33:40, 59:69)<br>caudal peduncle and fin (8,9, 41:58, 70:105)
